# Degenerative Changes in the Cervical Spine Are More Common in Middle-Aged Individuals with Thalidomide Embryopathy than in Healthy Controls

**DOI:** 10.1371/journal.pone.0155493

**Published:** 2016-05-13

**Authors:** Shadi A. Ghassemi Jahani, Aina Danielsson, Rana Ab-Fawaz, Hanna Hebelka, Barbro Danielson, Helena Brisby

**Affiliations:** 1 Department of Orthopaedics, Kungälv Hospital, 442 41, Kungälv, Sweden; 2 Department of Orthopaedics, Sahlgrenska University Hospital, 413 45, Gothenburg, Sweden; 3 Department of Radiology, Section of Musculoskeletal Radiology, Sahlgrenska University Hospital, 413 45, Gothenburg, Sweden; 4 Department of Radiology, Queen Silvia Children Hospital, 413 19, Gothenburg, Sweden; 5 Department of Orthopaedics, Institute of Clinical Sciences, Sahlgrenska Academy, University of Gothenburg, 413 45, Gothenburg, Sweden; 6 Department of Radiology, Institute of Clinical Sciences, Sahlgrenska Academy, University of Gothenburg, 413 45, Gothenburg, Sweden; University of Crete, GREECE

## Abstract

**Background:**

Thalidomide was used as a sedative drug for pregnant women in the 1950–60:s and resulted in children born with thalidomide embryopathy (TE), including upper limb malformations. These may alter the motion pattern of the cervical spine by the use of head/shoulder and mouth grip.

**Aims:**

To compare degenerative changes in the cervical spine in TE individuals with healthy controls (CTR).

**Methods and Procedures:**

Twenty-seven middle-aged TE individuals and 27 age- and gender-matched CTR were examined by cervical spine MRI. The presence of malformations, disc herniation(s), osteophytes, nerve and medullary compression and the degree of disc degeneration (DD) were evaluated.

**Outcomes and Results:**

Significantly higher degree of DD was seen in the TE group compared with the controls (p<0.001). Similar frequencies of disc herniation and disc space narrowing were observed in the two groups, but more foraminal narrowing was seen in the TE group (p = 0.002). DD was observed relatively frequently at all cervical levels in the TE group, however, mainly at the two lower levels in the CTR.

**Conclusions and Implications:**

Middle-aged individuals with TE have a higher frequency of degenerative changes in the cervical spine than controls, possibly caused by an altered load on the cervical spine.

## Introduction

In the mid-1950:s thalidomide was introduced as a sedative medication and it became popular amongst pregnant women worldwide [1, 2]. This substance was found to have teratogenic effects, however, and at the beginning of the 1960:s many children with multiple malformations were born as a result of its use [3]. These children with so-called thalidomide embryopathy (TE), presented with varying degrees of malformations of many organ systems mostly affecting the extremities [4, 5]. Other orthopaedic manifestations have also been described, including spinal defects such as block vertebrae, spondylolysis, scoliosis, malformations of the intervertebral disc, and dysgenesia of the sacrum [6, 7].

Today, thalidomide is mostly used for treatment of multiple myeloma and erythema nodusom leprosum [8, 9]. However, due to availability of the drug in combination with failure to inform about the side effects of the drug, children with TE are still being born throughout the world [10, 11].

Degeneration of the cervical spine is known to increase not only with age [12] but also due to other factors such as an increased mechanical load of the spine and genetic predisposition [13, 14]. Degenerative changes may cause clinical symptoms if neural structures are affected, as may occur in disc herniation and foraminal or central stenosis, but they may also play a role in patients with unspecific neck pain. The degenerative process is mostly slow and may not lead to symptoms until severe changes or compromise of nerves occur. [15, 16]

In TE individuals, the upper limb malformations often necessitate help with grip function through the mouth or by extreme movement (forward flexion and sometimes rotation) of the head, which leads to altered cervical spine movements. Theoretically, this may lead to degeneration of the cervical spine at an earlier age in patients with TE than in others.

The aim of this study was to investigate the presence of malformations and degenerative changes in the cervical spine in a group of middle-aged TE individuals and to compare the degenerative changes and possible nerve compromise with that in an age- and gender-matched control group.

## Material and Methods

### Study group

#### Thalidomide Embryopathy individuals

Thirty-one individuals with thalidomide embryopathy, (TE) who were contacted through The Swedish Thalidomide Society agreed to participate in a comprehensive multi-disciplinary study of TE individuals [17]. The present investigation of cervical spine pathology was one of the sub-studies. At the time of the investigation, the association had 108 members. A separate invitation for undergoing an MRI of the cervical spine was sent out and 30 of the 31 of the group in the multi-disciplinary study agreed to participate. However, two individuals withdrew from participation due to claustrophobia and one had intracranial metal clips that precluded any MRI investigation. Finally, 27 individuals with TE were included in the study. The mean age of the participants was 46 years (range 45–50) and 11 (41%) were women (Table 1).

**Table 1:** Age and gender demographics.

Variable	TE (n = 27)	CTR (n = 27)	p-value
Gender, n (%)			
■ Male	16 (59)	15 (57)	0.452
■ Female	11 (41)	12 (44)	
Age, Mean (SD)/(Range)	46.2 (1.0)/(45–50)	46.1 (1.9)/(40–50)	0.498

TE: Thalidomide Embryopathy

CTR: Control group

The diagnosis of TE individuals of the Swedish association was made in the early 1960:s, shortly after their birth, by a single paediatrician [18, 19]. The TE diagnosis were based on confirmed usage of thalidomide by the mothers, the presence of bilateral phocomelia and/or amelia (as later described by Castilla et al.) [10], and /or by malformation of other organs, considered specific for TE. The latter included malformations of the ears and eyes as well as other malformations [20–24]. Eighty-five per cent of the TE individuals in the investigated group had hand anomalies. All twenty-seven had some kind of functional arm/arms, i.e. some type of grip function, although only nine individuals had a bilateral proper pincer grasp and some had very short upper limbs (Table 2)

**Table 2:** Description of the TE individuals’ upper and lower extremities limb reduction/anomalies.

Participants	Upper extremities	Lower extremities
1	Bilateral thumb malformations/ or absence	0
2	Bilateral shoulder anomalies	0
3	Bilateral thumb malformations/ or absence	PFFD with major malformations
4	Bilateral club hands *	0
5	Bilateral thumb malformations/ or absence	0
6	Bilateral thumb malformations/ or absence	PFFD with major malformations
7	Bilateral club hands *	Anomaly of both hips
8	Bilateral thumb malformations/ or absence	0
9**	0	0
10	Bilateral phocomelia	Malformations of toes
11	Bilateral club hands *	0
12	Bilateral club hands*	PFFD with major malformations
13	Bilateral wrist malformations	PFFD with major malformations
14	Bilateral thumb malformations/ or absence	0
15**	0	0
16	Bilateral phocomeliae	0
17	Bilateral club hands *	0
18	Bilateral thumb malformations/ or absence	0
19	Bilateral thumb malformations/ or absence	0
20	Bilateral shoulder anomalies	0
21**	0	0
22	Bilateral thumb malformations/ or absence	0
23	Bilateral club hands*	PFFD with major malformations
24	Short forearm at one side	0
	Bilateral thumb malformations/ or absence	
25	Bilateral thumb malformations/ or absence	0
26	Bilateral club hands*	0
27	Bilateral thumb malformations/ or absence	0

* Club hands: absence or hypoplasia of radius in forearms and different grade of radial bowing of the forearms with absence or hypoplasia of the thumbs

** Patient # 9; Duane Syndrom with (an abnomal ocular motility), bilateral external ear malformations with hearing deficit and bilateral facial palsy

Patient # 15; Duane syndrome with bilateral external ear malformations with hearing deficit and facial palsy at the right side. Patient #21; Duane syndrome with bilateral, external ear malformations and hearing deficit. PFFD: Proximal Focal Femoral Deficiency, where the femur, including the hip joint, is reduced in length from nearly normal to barely non-existing. Major malformations include tibia or fibula hypo- or aplasia or foot deformities.

All TE individuals underwent a clinical examination by one single orthopaedic surgeon, including inspection regarding scoliosis and other obvious deformities. Neurological examinations of the upper and lower extremities were performed and patient-reported symptoms of radiating pain in any of the extremities were recorded. Cervical spine mobility, flexion, extension, side rotation, and side flexion, were measured using the Myrin goniometer^®^ (LIC Rehab Care, Sweden) [25] and the individuals were asked if any of these movements provoked pain.

#### Control group (CTR)

The control group consisted of a gender- and age-matched group of individuals. They were selected using the patient archiving and communication system (PACS) at the Department of Radiology, Sahlgrenska University Hospital in Gothenburg, Sweden, from patients in whom MRI of the whole spine had been performed between 2003 and 2014 (without any specific referral inquiries regarding cervical pathology). The exclusion criteria used were symptoms listed at the referral that might be caused by pathology of the cervical spine, such as neck pain or brachialgia, a previous history of cervical spine disease or earlier interventions performed in the cervical region.

The mean age of the individuals in the CTR (n = 27) was 46 years (range 40–50 years) and 12 (44%) were women (Table 1).

### MRI investigations

MRI of the cervical spine was performed on 20 of the TE group at the Department of Radiology of Sahlgrenska University Hospital and it was performed on the remaining seven individuals with TE at their regional hospital, for practical reasons. The protocol used was identical for all examinations.

#### MRI protocol

The MRI examinations in the TE group were carried out in 2008 on three different 1.5T (Tesla) scanners: 23 (85%) using a Philips Medical Systems scanner (Intera), 3 (11%) using a Siemens scanner (Symphony), and in one (4%) using a GE Medical Systems scanner (SignaExcite). A standard protocol for the cervical spine was used, including sagittal T1-weighted turbo spin echo (T1W-TSE) sequence with repetition and echo time (TR/TE) of 400–597/8–10 milliseconds (ms) and T2W-TSE sequence with TR/TE of 3500–4920/104–120 ms. Axial images included were T2W gradient-recalled echo (GRE) sequence with TR/TE of 537–960/14–17ms and T2W-TSE images with TR/TE of 1100–2500/100–120 ms. For all sequences, field of view (FOV) was 10cm, slice thickness 3–6 mm and matrix size 576 x 576 to 512 x 512.

Since the MRI examinations for the CTR were performed based on various clinical indications, few patients had an axial image included—which is why only sagittal images were evaluated. Sagittal T1W and either sagittal T2W or short tau inversion recovery (STIR) sequences were included for all individuals with 25 of the 27 (93%) examinations performed on 1.5T Philips Medical Systems scanners (Achieva/Intera). The sagittal T1W-TSE and T2W-TSE sequences were performed almost identically as for the TE group. STIR imaging was performed with TR/TE of 1776–4000/60–90 ms and inversion time (TI) of120–170 ms.

#### MRI evaluation

In each subject, C2 to C7 were evaluated. Each disc level was assessed for the degree of degeneration according to Pfirrmann [26], narrowing of disc space, presence of anterior or posterior osteophytes, anterior or posterior disc bulge, disk herniation, foraminal stenosis, anterior compression of the dural sac and presence of malformations such as block vertebrae. [27–29]. At least 25% loss of height of a single disc relative to adjacent normal levels was defined as disc space narrowing. Disc bulge was assessed on sagittal T1W and T2W images, and referred to a diffuse protrusion of the disc by more than 2 mm away from the vertebral margins anteriorly or posteriorly [27]. Disc herniation was defined as a localized/focal protrusion of the disc more than 2 mm from the vertebral margins when, in at least one plane, any distance between the edges of the disc material beyond the disc space was greater than the distance between the edges of the base. The direction of disc herniation (central, right or left paracentral, or right or left foraminal) was noted. Foraminal stenosis was assessed on sagittal T1W images and defined as obliteration of the intraforaminal fat. The degree of spinal cord impingement/compression adjacent to a site of disc bulging and presence of anterior or posterior osteophytes was also evaluated.

In the TE group both sagittal and axial sequences were used for assessment of disc bulge/ herniation and foraminal stenosis whereas only sagittal sequences were used in the CTR due to the lack of axial sequences. All other parameters evaluated were assessed in an identical manner.

All evaluations were analyzed first for occurrence of a specific variable in each subject, and thereafter analyzed for the total number of levels for all 27 individuals of each group, i.e. 27 subjects with five levels each makes a total of 135 levels.

The parameters were evaluated independently by one resident and one senior radiologist at the Sahlgrenska University Hospital. Blinded regarding the first evaluation, the resident repeated the evaluation process after four weeks.

### Ethics

The study was approved by the local human research ethics committee at the University of Gothenburg, Ö 556–03.

The prospectively included TE individuals were provided with written and verbal information about the procedure of the investigation and the study and all signed confirmed consent. The control group MRIs were retrospectively selected from the patient archiving and communication system at the hospital radiology department to match the age and gender for the TE group and their MRI investigations unidentified before analyzed, therefore consent was not applicable.

### Statistical Analysis

For comparisons between groups, Fisher’s exact test was used for dichotomous variables and Mantel-Haenszel chi-square exact test was used for ordered categorical variables. Chi-square exact test was used for unordered categorical variables and Mann-Whitney U-test was used for continuous variables. The kappa statistic (κ) was used to assess the inter- observer and intra-observer agreement. All tests were two-tailed and conducted at the five per cent significance level. The statistical analyses were performed using SPSS software version 19.

## Results

### Clinical examination and malformations

None of the individuals in the TE group had any signs of scoliosis at the clinical examination, and only one had a radiological detectable malformation: a block vertebra of the C7-T1 vertebrae.

The Myrin goniometer was attached to the head of each person and was read without any difficulties. None of the TE individulas experienced pain with any of the movements. Mean range of motion (ROM) of the cervical spine in the TE group was 28° (SD 8.6, range 12–50) and 30° (SD 8.5, range 20–50) for flexion to the left and right side, respectively. Mean ROM in extension was 46.5° (SD 14, range 15–75) and mean ROM in flexion was 44° (SD 16, range 0–80). Mean rotation of the head to the right was 52° (SD 15, range 20–80) and mean rotation to the left was 54° (SD 15, range 25–80).

All TE individuals showed normal muscle reflexes in the present upper and lower extremities, when it was possible to examine them. There were no motor or sensory deficits detected in any of the TE subjects.

### Degenerative changes in the cervical spine in TE individuals compared to controls

In the TE group, 24 of the 27 individuals (89%) had degeneration of at least one disc in the cervical region as compared with 18 of 27 (67%) in the CTR (p< 0.001). Ten of the 27 individuals within the TE group had degenerative signs, to different degrees, in as many as four motion segments, while no one had more than three degenerated segments in the CTR (p<0.001) (Table 3).

**Table 3:** Individuals with disc degeneration, central or foraminal narrowing both sides.

**Number of degenerated discs n(%)**			
	TE* **(n = 27)**	CTR** **(n = 27)**	**p-value**
0	3 (11)	9 (33)	
1	2 (7)	3 (11)	
2	6 (22)	11 (41)	< 0.001
3	6 (22)	4 (15)	
4	10 (37)	0 (0)	
**Number of segment with central canal narrowing n(%)**			
	TE* **(n = 27)**	CTR** **(n = 27)**	**p-value**
0	21 (78)	18 (67)	
1	1 (4)	5 (19)	
2	3 (11)	3 (11)	
3	0	1 (4)	0.905
4	1 (4)	0	
5	1 (4)	0	
**Number of affected foramens on any side n(%)**†			
	TE* **(n = 27)**	CTR** **(n = 27)**	**p-value**
0	3 (11)	15 (56)	
1	8 (30)	2 (7)	
2	3 (11)	4 (15)	
3	4 (15)	5 (19)	
4	3 (11)	1 (4)	0.002
5	2 (7)	0	
6	3 (11)	0	
7	0	0	
8	1 (4)	0	
9	0	0	
10	0	0	

* TE Thalidomide Embryopathy.

** CTR: Control.

^†^ All 10 foramina, left and right.

The parameters were evaluated independently by one resident and one senior radiologist at the Sahlgrenska University Hospital. Blinded regarding the first evaluation, the resident repeated the evaluation process after four weeks.

#### Ethics

The study was approved by the local human research ethics committee, Ö 556–03.

#### Statistical analysis

For comparisons between groups, Fisher’s exact test was used for dichotomous variables and Mantel-Haenszel chi-square exact test was used for ordered categorical variables. Chi-square exact test was used for unordered categorical variables and Mann-Whitney U-test was used for continuous variables. The kappa statistic (κ) was used to assess the inter- observer and intra-observer agreement. All tests were two-tailed and conducted at the five per cent significance level. The statistical analyses were performed using SPSS software version 19.

**Fig 1:**
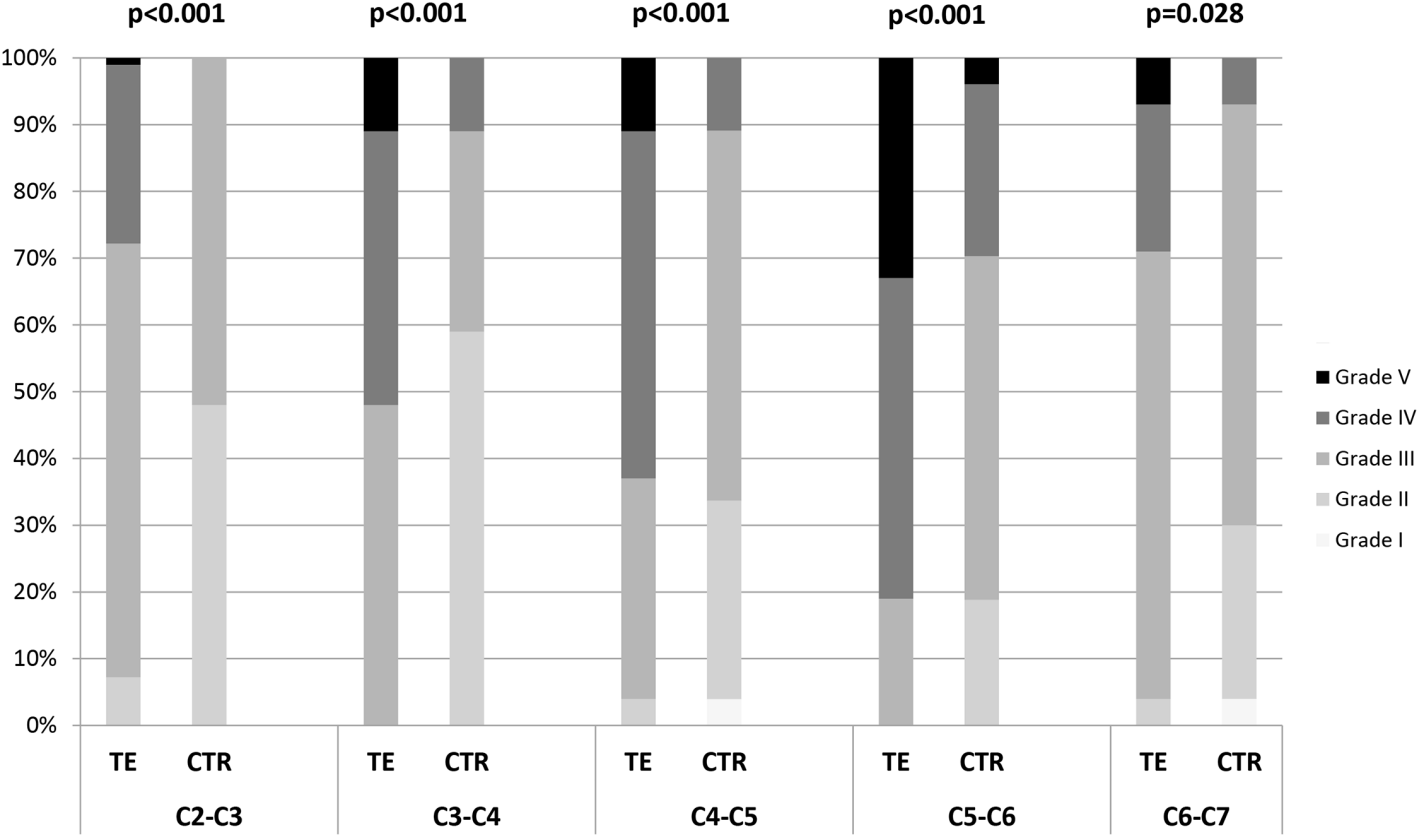
Disc degeneration evaluated by Pfirrmann classification on MRI. Pfirrmann grading (I-V) of all cervical discs in 27 individuals with thalidomide embryopathy (TE), and 27 aged- and gender matched control subjects (CTR). Each cervical level (C2-C3-C6-C7) is presented separately and Fisher Exact Test was used for comparison between the two groups. Significant differences between the two groups were seen at all levels (p-values in the figure).

Classification of disc degeneration according to Pfirrmann and comparisons between the TE group and the CTR are presented in Fig 1 and Table 4. For both the TE group and the CTR, degenerative changes were common in the lower levels of the cervical spine (C5-C6 and C6-C7), however for the TE group degenerative changes, were often also seen in the upper segments. The TE grop showed significantly higher Pfirrmann grades at all the five evaluated levels compared with the CTR.

**Table 4:** Comparison of degenerative findings in all cervical segments between the TE group and the CTR group, n (%).

Disc signal (Pfirrmann)(n = 135)	TE n(%)	CTR n(%)	p-value
I	0 (0)	2 (2)	
II	4 (3)	49 (36)	
III	62 (46)	68 (50)	<0.001
IV	51 (38)	15 (11)	
V	18 (13)	1 (1)	
**Disc hernia (n = 135)**			
Yes	9 (7)	4 (3)	0.155
**Disc height reduction (n = 135)**			
0–25%	120 (89)	122 (90)	
26–50%	10 (7)	11 (8)	0.509
> 50%	5 (4)	2 (1)	
**Osteophytes (n = 135)**			
Yes	64 (47)	32 (24)	<0.001
**Disc protrusion (n = 135)**			
Yes	39 (29)	12 (9)	<0.001
**Foramen (R + L, n = 270)**			
No compromise	196 (73)	241 (89)	
Narrowing, normal nerve root	33 (12)	24 (9)	< 0.001
Narrowing, nerve root compressed	41 (15)	5 (2)	

TE: Thalidomide Embryopathy

CTR: Control group

### Disc pathology and osteophytes

Disc herniation was seen in nine discs (7%) in the TE group compared with four (3%) in the controls. Disc space narrowing of over 50% was observed in five discs (4%) in the TE group and in two discs (1%) in the CTR (Table 4). In the TE group 64 (47%) of all 135-motion segments presented with osteophytes, as opposed to 32 (24%) in the CTR (p<0.001) (Table 4).

**Fig 2:**
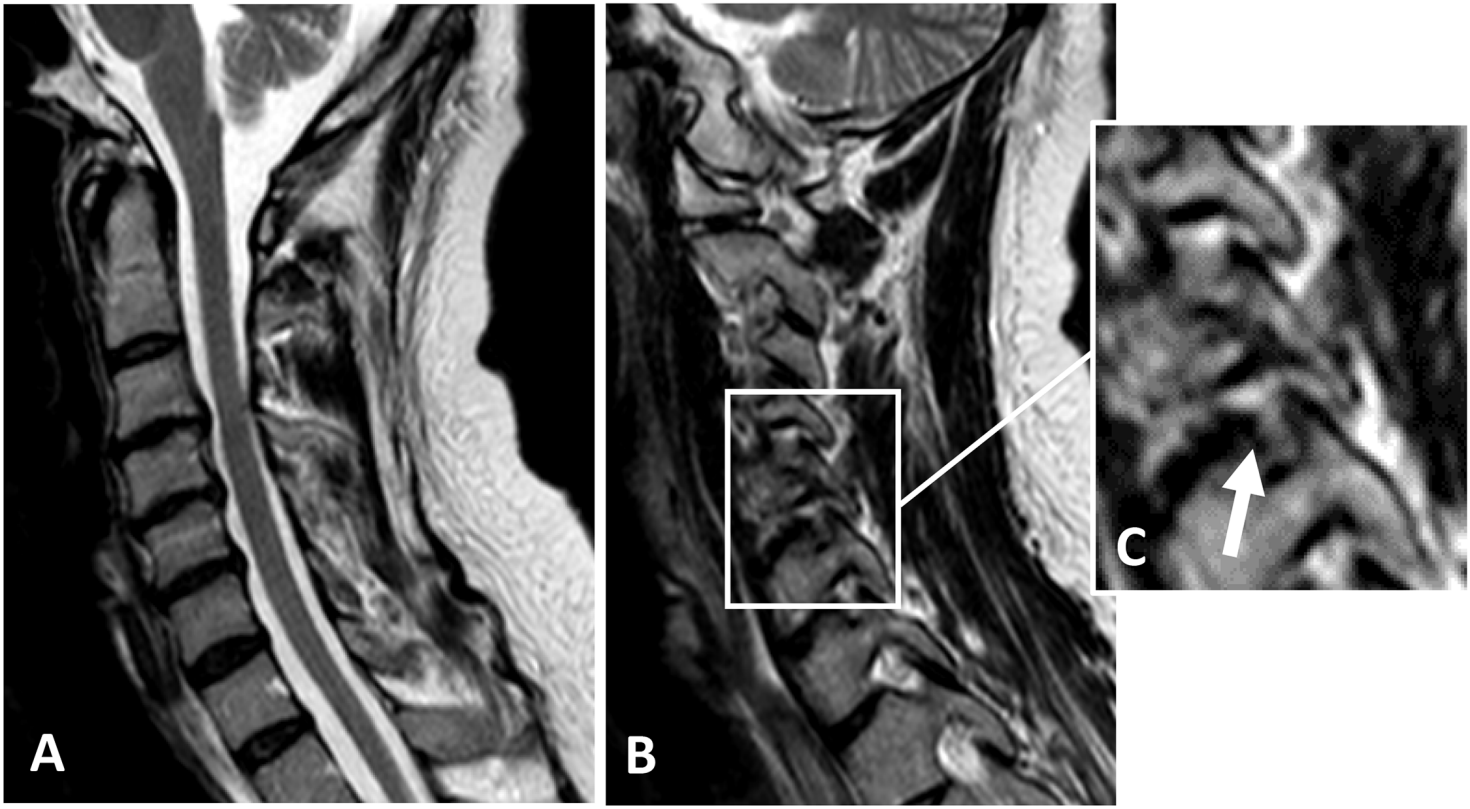
MRI image of cervical spine in TE. MRI of the cervical spine for one of the TE patients where moderate disc degeneration was seen at C3-4, C4-5 and C5-6 level (A). Foraminal narrowing with nerve compromise can be seen here at level C5-6 on the right side (B and enlargement C, with arrow pointing at the disc/bulge/nerve compromise).

In Fig 2 the sagittal image on MRI from cervical spine in one participant with TE is shown.

### Foraminal and central stenosis

Twenty-four subjects in the TE group had at least one affected foramen, at one or more segments, as compared with 12 in the control group. The number of affected foramina per individual was also higher in TE group (p = 0.002) (Table 3). When looking at the foraminal changes for all 270 foramina per group independently, there was also a significantly higher frequency of foraminal narrowing in the TE group, than in the CTR. For the TE group, 41 (15%) showed foraminal narrowing with nerve root compression and 33 (12%) showed narrowing without nerve root compression. The corresponding numbers for the CTR were 5 (2%) and 24 (9%), respectively (p<0.001) (Table 4).

### Inter- and intra-observer agreement

Inter- and intra-observer kappa scores regarding Pfirrmann grading were 0.8 and 0.6, respectively. The kappa-values for both types of agreement regarding disc herniation and foraminal stenosis on either side were 0.8–1.0 and 0.7–0.8, respectively, for disc protrusions. The inter- and intra-observer kappa values for disc space narrowing and presence of osteophytes were somewhat lower (0.3–0.5), and corresponding kappa values for spinal cord compression were 0.5–0.7.

## Discussion

Individuals with thalidomide embryopathy (TE) had increased frequencies of disc protrusion, foraminal narrowing, osteophytes, and disc signal decrease on MRI, relative to a healthy gender- and age-matched population. These findings suggest that there might be an earlier development of disc degeneration in individuals with TE.

People with TE often have several anomalies, including musculoskeletal malformations of the extremities. The present study was initiated as a multi-disciplinary follow-up of middle-aged adults with TE, and the present study of the cervical spine was one of the objectives. Despite the fact that a large number of studies on this patient group has been performed, no previous studies have addressed the question of whether TE patients have increased degenerative signs of the spine.

The present group of middle-aged TE individuals is a unique cohort providing an opportunity to investigate degeneration of the cervical spine in a group with a changed cervical motion pattern, including extra load of the cervical spine when performing tasks with the head in unusual positions. Examples of such tasks are to use the mouth to hold on to an article of clothing when dressing, the untying of shoes or holding an item between a short limb and the head, when lifting and carrying an item e.g. a mobile phone. Another example is to open a plastic bag with the teeth [30], which was described by some of the participants in TE group; however, the majority of the TE individuals used their upper limbs when performing different tasks, even though most often in modified ways.

When the findings from MRI of the TE group were compared with those from an age-matched control group, there was a larger proportion of degenerative findings in the TE group. This included disc changes, such as disc protrusion and disc signal changes, and osteophytes. These degenerative changes also caused a higher frequency of foraminal canal compromise in the TE group. Overall, the TE patients had few malformations of the cervical spine, only one with block vertebrae at C7 was found. Thus, no influences of malformation on the development on degenerative changes were likely. Since none of the individuals in the TE group presented any clinical symptoms of nerve root or medullary compromise at the time of investigation, these changes did not appear to be related to any major clinical problem.

There are studies suggesting increased disc degeneration of the cervical spine due to loading and changes of the sagittal alignment of the cervical spine in healthy populations [31]. An association has also been found between heavy-load bearing on the head and increased radiographic degenerative signs [32, 33]. This type of external load has also been suggested to simultaneously produce more stiffness and pain in the cervical spine [33]. The findings of increased degeneration on MRI in cervical spine in TE individuals might suggest that by altered loading of the cervical spine in different ways than normal e.g. repeated flexing of the head to one side, even if no particularly heavy weights are applied, might hasten the development of degenerative changes in the cervical spine.

Degeneration of the cervical spine is known to preferably affect the lower segments [12, 14] and degenerative findings were frequently seen in the three lower levels in the present study’s patients in both the TE and the CTR groups. However, in the TE group degenerative changes were frequently also seen at the upper levels, which one could argue might indicate that there is a more general effect on cartilage/intervertebral discs in TE individuals, which might be based on a genotype level. On the other hand, this finding might reflect the increased stress also on these upper levels, associated with an altered motion pattern.

In a previous report, we found that the frequency of osteoarthritis of the hip, 38%, and of the knee, 58%, was higher than previously published results from the general population. [17]. These findings are primarily considered to be caused by an altered load of these large joints, but of course a more direct effect on cartilaginous tissue by the thalidomide drug during embryogenesis cannot be ruled out.

The mechanisms behind the increased presence of cervical spine degeneration are, as with knee and hip joint osteoarthritis, unclear. However, the fact that few malformations of the cervical spine were seen, might speak in favor for the theory of increased degeneration induced by greater mechanical stress.

The major limitation of the present study was the low number of study subjects, however, with such a rare condition this must be considered a relatively large cohort. Moreover, the similarly sized control group, used for comparison with the TE individuals, selected from individuals with previous MRI cervical spine investigations were not ideal in terms of 1) no self-reported information about symptoms, 2) the exclusion of patients with second hand reported neck pain (which may have introduced a difference between controls and TE individuals; where no information on neck pain were collected) and 3) the lack of axial sequences on MRI. Despite the limited numbers of individuals, significant differences were observed between the two study groups.

Substantial or very good inter- and intra-observer agreement was found for the majority of MRI changes. The exceptions were the agreements for disc space narrowing and presence of osteophytes, which were found to be moderate to fair, and inter-observer agreement in terms of disc signal intensity, which was moderate. When analyzing the latter figure in detail, the majority of inconsistencies between the readers were between Pfirrmann grade 2 and 3, whereas there was strong agreement in terms of the other Pfirrmann grades.

## Conclusion

Taken together, individuals with TE show the same signs of degeneration in the cervical spine, as seen in the general population, but to a greater extent and distributed within the whole cervical spine. These findings are of particular interest for individuals with TE malformations of the extremities, but possibly also for other groups with limb malformations, since altered load of the cervical spine may be the reason behind this. To explore the effect of altered load further, studies on larger patient groups with other upper limb defects would be desired. These findings may also be of relevance for the understanding of the development of degeneration in individuals with professions or activities that cause a high degree of stress or load on the cervical spine. If these changes lead to higher risk of future central or foraminal stenosis symptoms or axial pain problems is unclear.
